# Therapeutic Duplication in Taiwan Hospitals for Patients With High Blood Pressure, Sugar, and Lipids: Evaluation With a Mobile Health Mapping Tool

**DOI:** 10.2196/11627

**Published:** 2020-07-27

**Authors:** Wei-Chih Kan, Shu-Chun Kuo, Tsair-Wei Chien, Jui-Chung John Lin, Yu-Tsen Yeh, Willy Chou, Po-Hsin Chou

**Affiliations:** 1 Department of Nephrology Chi Mei Medical Center Tainan Taiwan; 2 Department of Biological Science and Technology Chung Hwa University of Medical Technology Tainan Taiwan; 3 Department of Ophthalmology Chi Mei Medical Center Tainan Taiwan; 4 Department of Optometry Chung Hwa University of Medical Technology Tainan Taiwan; 5 Medical Research Chi Mei Medical Center Tainan Taiwan; 6 USA Sports Medicine Sherman Oaks, CA United States; 7 Medical School St George’s, University of London London United Kingdom; 8 Department of Physical Medicine and Rehabilitation Chiali Chi Mei Hospital Tainan Taiwan; 9 Department of Physical Medicine and Rehabilitation Chung Shan Medical University Taichung Taiwan; 10 Department of Orthopedics and Traumatology Taipei Veterans General Hospital Taipei Taiwan; 11 School of Medicine National Yang-Ming University Taipei Taiwan

**Keywords:** duplicate medication, mHealth, hypertension, high blood sugar, high blood lipid

## Abstract

**Background:**

Cardiovascular disease causes approximately half of all deaths in patients with type 2 diabetes. Duplicative prescriptions of medication in patients with high blood pressure (hypertension), high blood sugar (hyperglycemia), and high blood lipids (hyperlipidemia) have attracted substantial attention regarding the abuse of health care resources and to implement preventive measures for such abuse. Duplicative prescriptions may occur by patients receiving redundant medications for the same condition from two or more sources such as doctors, hospitals, and multiple providers, or as a result of the patient’s wandering among hospitals.

**Objective:**

We evaluated the degree of duplicative prescriptions in Taiwanese hospitals for outpatients with three types of medications (antihypertension, antihyperglycemia, and antihyperlipidemia), and then used an online dashboard based on mobile health (mHealth) on a map to determine whether the situation has improved in the recent 25 fiscal quarters.

**Methods:**

Data on duplicate prescription rates of drugs for the three conditions were downloaded from the website of Taiwan’s National Health Insurance Administration (TNHIA) from the third quarter of 2010 to the third quarter of 2016. Complete data on antihypertension, antihyperglycemia, and antihyperlipidemia prescriptions were obtained from 408, 414, and 359 hospitals, respectively. We used scale quality indicators to assess the attributes of the study data, created a dashboard that can be traced using mHealth, and selected the hospital type with the best performance regarding improvement on duplicate prescriptions for the three types of drugs using the weighted scores on an online dashboard. Kendall coefficient of concordance (W) was used to evaluate whether the performance rankings were unanimous.

**Results:**

The data quality was found to be acceptable and showed good reliability and construct validity. The online dashboard using mHealth on Google Maps allowed for easy and clear interpretation of duplicative prescriptions regarding hospital performance using multidisciplinary functionalities, and showed significant improvement in the reduction of duplicative prescriptions among all types of hospitals. Medical centers and regional hospitals showed better performance with improvement in the three types of duplicative prescriptions compared with the district hospitals. Kendall W was 0.78, indicating that the performance rankings were not unanimous (Chi square_2_=4.67, *P*=.10).

**Conclusions:**

This demonstration of a dashboard using mHealth on a map can inspire using the 42 other quality indicators of the TNHIA by hospitals in the future.

## Introduction

Cardiovascular disease causes approximately half of all deaths in patients with type 2 diabetes [1,2]. At the population level, an increasing proportion of all cardiovascular events can be attributed to the presence of diabetes [3]. Many epidemiological studies have shown a direct relationship between the levels of blood pressure, glycemia, low-density lipoprotein-cholesterol, and complications of diabetes [4-7]. However, the therapeutic duplication of medication in patients with high blood pressure, high blood sugar, and high blood lipids has attracted substantial attention to prevent the abuse of health care resources.

Duplicative prescriptions refer to situations in which patients receive redundant medications for the same condition from two or more sources [8] such as doctors, hospitals [9,10], multiple providers [11], or as a result of the patient’s wandering, in which they move from hospital to hospital for the same condition [12]. Doctor (or hospital) shopping (ie, seeking care from multiple doctors without professional referral for the same or similar conditions) is common in Asia [9,13]. According to Takahashi et al [13], approximately 5.8% of outpatients in Japan self-reported that they visited multiple medical facilities for treatment of the same conditions.

The prevalence of duplicative prescriptions is estimated at 7.4% in Japan [13], which is higher than the rate of 0.43% in Taiwan [14] due to the use of different definitions regarding the Anatomical Therapeutic Chemical (ATC) classification in which the first three five digits are used in Japan and Taiwan, respectively. The management criteria (or tolerance thresholds) of duplicative prescriptions in Taiwan are set at 0.5805%, 0.4273%, 0.5934%, 1.2866%, and 0.9214% for a medical center, regional hospital, local hospital, clinic, and pharmacy, respectively [15], leading the Taiwan National Health Insurance Administration (TNHIA), which operates under the Ministry of Health and Welfare, to strongly express concern about the practice of duplicative prescriptions.

From the perspective of therapeutic safety and excess expenditures, patients who receive medical care from different medical facilities are more likely to receive duplicative prescriptions and suffer adverse drug reactions [9,16-18]. The prevalence of duplicative prescriptions was defined by the TNHIA as the practice of a patient who receives identical medications (based on the first five digits of the ATC) from an identical facility (eg, hospital or clinic) for a period of several overlaid days (ie, total duplicative days/total prescriptive days in a specific period) [14]. A total of 12 indicators of duplicative prescriptions (ie, types of drugs used in the treatment of diseases) have been included and announced quarterly by the TNHIA [19] to help health care providers facilitate management so as to reduce the rate of duplicative prescriptions.

Furthermore, increasing the transparency of hospitals is a requirement to improve administration with regard to patient safety [20-22]; therefore, disclosing the performance of hospitals in effectively controlling duplicative prescriptions to the public is required. If a hospital wants to achieve improvement in patient safety, inspection of a publicly available quality reporting system is essential. Indeed, transparency has been demonstrated as the most powerful driver of health care improvement [23].

By searching for the key words “duplicative prescriptions” on PubMed on April 22, 2020, only one paper [13] was retrieved that reported duplicative prescriptions using social network analysis (SNA). We did not find any study proposing an appropriate method to decrease the number of duplicative prescriptions. That is, when using SNA for interpreting duplicative prescriptions [6], the management perspective is limited in identifying key viewpoints that should be considered in dealing with the duplicative prescription issue.

The SNA approach [24-27] is used to define facilities as the “nodes” of a prescribing network connected to another node (eg, a square box) with a patient duplicative prescription represented as an edge (eg, a connecting arrow). For example, a string of “4 3 1” denotes that node 4 prescribed a duplicative medication via a patient (with a weight of 1) to node 3 using the displayed graphical presentation in which node 4 is connected to node 3 with an arrow.

The objectives of the present study were to (1) assess the attributes of the study data using scale quality indicators, (2) create a dashboard (ie, a control panel on a webpage that collates visual information about an issue or a topic that can be manipulated by readers themselves [28] and can be traced using mobile health [mHealth]), and (3) select the hospital type that shows the best performance in improving duplicate prescriptions of three types of medications (antihypertension, antihyperglycemia, and antihyperlipidemia) using the weighted scores across the types of hospital and performance percentages on an online dashboard. Finally, the Kendall coefficient of concordance (*W*) [29,30] was used to evaluate the unanimity of the performance rankings.

## Methods

### Study Data

All ratio data for the three types of duplicative prescriptions on the website of TNHIA [19] were downloaded on April 7, 2018 for all registered hospitals in Taiwan. The inclusion criteria were the period from 2010 to 2016 and data recorded in the quarter. Data from a total of 25 quarters (ie, from the third quarter of 2010 to the third quarter of 2016) were included. The exclusion criterion was incomplete ratio data in these 25 quarters. Three types of hospitals, including medical centers, regional hospitals, and district hospitals, were classified and compared. A total of 408, 414, and 359 hospitals were included as study samples for antihypertension, antihyperglycemia, and antihyperlipidemia medications, respectively (Table 1). All data regarding duplicative prescriptions were determined by the ATC classification using the first five digits according to the guideline in Taiwan.

**Table 1:** Descriptive statistics of hospitals included in the study.

Drug and hospital type		Taipei, n (%)	North, n (%)	Central, n (%)	South, n (%)	Kao-Pin, n (%)	East, n (%)
**Antihypertension**							
	Medical Center (N=20)	7 (35)	2 (10)	4 (20)	3 (15)	3 (15)	1 (5)
	Regional Hospital (N=77)	11 (14)	17 (22)	17 (22)	14 (18)	15 (19)	3 (4)
	District Hospital (N=305)	29 (10)	62 (20)	86 (28)	40 (13)	76 (25)	12 (4)
	Total (N=402)	47 (12)	81 (20)	107 (27)	57 (14)	94 (23)	16 (4)
**Antihyperglycemia**							
	Medical Center (N=20)	7 (35)	2 (10)	4 (20)	3 (15)	3 (15)	1 (5)
	Regional Hospital (N=79)	11 (14)	18 (23)	16 (20)	16 (20)	15 (19)	3 (4)
	District Hospital (N=308)	29 (9)	63 (20)	88 (29)	47 (15)	69 (22)	12 (4)
	Total (N=407)	47 (12)	83 (20)	108 (27)	66 (16)	87 (21)	16 (4)
**Antihyperlipidemia**							
	Medical Center (N=20)	7 (35)	2 (10)	4 (20)	3 (15)	3 (15)	1 (5)
	Regional Hospital (N=77)	11 (14)	17 (22)	16 (21)	16 (21)	14 (18)	3 (4)
	District Hospital (N=257)	27 (11)	54 (21)	74 (29)	31 (12)	60 (23)	11 (4)
	Total (N=354)	45 (13)	73 (21)	94 (26)	50 (14)	77 (22)	15 (4)

### Assessing the Quality of Data

Good data quality is necessary to ensure acceptable reliability and validity [31,32].

Therefore, before analysis, the quality of the data was assessed to ensure compliance with responses that may be producible and predictable in similar studies using the following metrics.

#### Reliability

The reliability (ie, Cronbach α) should be greater than .70 [33].

#### Dimension Coefficient

The dimension coefficient [34] indicates the strength of unidimensionality, defined as Z/(1+Z), where Z=(a1/a2)/(a2/a3) and the values of a1, a2, and a3 are the eigenvalues of the first three principal components of a scale. The dimension coefficient ranges from 0 to 1; a value greater than 0.67 indicates a unidimensional scale [34].

#### Convergent Validity

Cronbach α tends to be overestimated. Therefore, it is recommended to rely more on convergent validity (or average variance extracted) and composite reliability values [35] as an assessment of reliability. Convergent validity can be computed as follows:


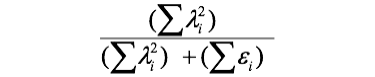
 (**1**)

Where λ is the item loading to the construct domain, λ^2^ indicates the communality to the factor, and denotes the measurement error.

#### Construct Reliability

Construct reliability is also called component reliability or composite reliability, which is expressed by the following formula:


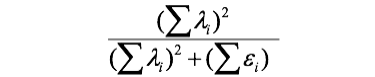
 (**2**)

where λ and ε are defined similarly to Equation 1.

### Building Online Dashboards on a Map

**Figure 1:**
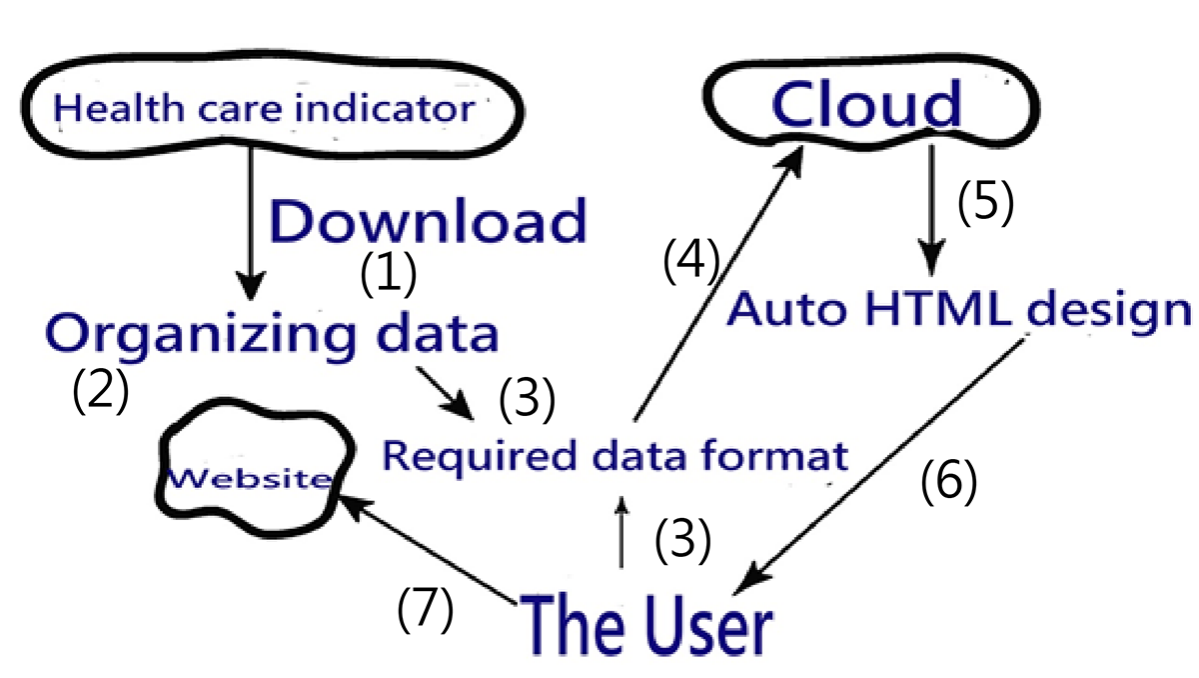
Flowchart made on a dashboard. All processes are described in detail in Multimedia Appendix 1.

Figure 1 shows the flowchart of cloud computation to build a quality report card on Google Maps based on quality indicators for data downloaded from the TNHIA website. After organizing the data to fit the required format for uploading, a user can immediately obtain the hypertext markup language (HTML) from the cloud computation through the following three steps: (1) upload data, (2) perform cloud computation, and (3) show an HTML page that can be downloaded for personal use or public navigation on the website. Interested readers are recommended to view the video demonstrating this process in Multimedia Appendix 1.

### Dashboard Features

The dashboard comprises the following five features: (i) the growth/share matrix of the Boston Consulting Group (BCG) on the map (ie, growth trend on the Y-axis and share on the X-axis) [36,37]; (ii) three traffic light color-coded clusters, which denote the degree of growth/share performance as excellent, fair, and poor; (iii) four quadrants represented by mascots (ie, dogs, question marks or problem children, stars, and cash cows) [37]; (iv) bubbles with a size proportional to product momentum (ie, duplicative prescription ratios in this study); and (v) a control area plotted by the 95% CI (ie, 2 SDs on the two axes).

**Figure 2:**
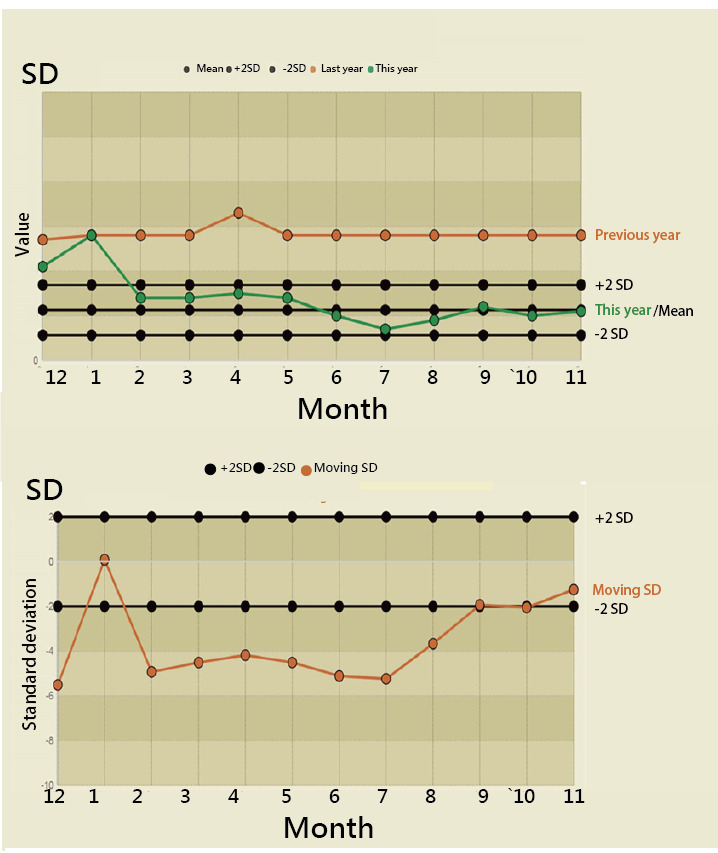
Comparison of traditional control chart (top) and moving average control chart (bottom, also see Multimedia Appendix 2) used in this study.

The growth (on the Y-axis, implying the trend based on recent time points) is determined by the trend via moving the control chart forward to the previous 12 months so that 24 data points yield 12 moving SDs (eg, datasets {–1,–1,–1,–1,–1,–1,–1,–1,–1,–1,–1,1} and {2,2,2,2,2,2,2,2,2,2,2,4} yield an identical correlation coefficient of 0.48 with the time series for 1 to 12), and the share (on the X-axis, indicating the accumulated momentum based on the past) is computed by the mean of the moving SDs (Figure 2 and Multimedia Appendix 2) through which the BCG growth/share matrix can be constructed by the four quadrants on Google Maps (eg, datasets {–1,–1,–1,–1,–1,–1,–1,–1,–1,–1,–1,1} and {2,2,2,2,2,2,2,2,2,2,2,4} yield different momentums of –0.83 and 2.17 across the 12 time points). The study datasets are shown in Multimedia Appendix 3.

### Examples for the Four Quadrants on a Dashboard

The following is a representative algorithm for locating the performance of hospitals on the four quadrants of a dashboard:

- Quadrant I: the dataset {2,2,2,2,2,2,2,2,2,2,3,4} using the moving control chart forward to the previous 12 months shows continuously increasing growth (ie, y=0.63) with a positive share (ie, x=2.25).
- Quadrant II: the dataset {–1,–1,–1,–1,–1,–1,–1,–1,–1,–1,1,1} shows preparedly increasing growth (ie, y=0.65) with a negative share (ie, x=–0.67).
- Quadrant III: the dataset {–1,–1,–1,–1,–1,–1,–1,–1,–1,–1,–2,–3} shows good performance in controliing duplicative prescriptions with respect to growth (ie, y=–0.63) with a negative share (ie, x=–1.25).
- Quadrant VI: the dataset {2,2,2,2,2,2,2,2,2,2,1,–1} indicates a decrease in growth (ie, y=–0.60) when the share is still positive (ie, x=1.67).

### Selecting the Best-Performing Hospital Types in the BCG Growth/Share Matrix

**Figure 3:**
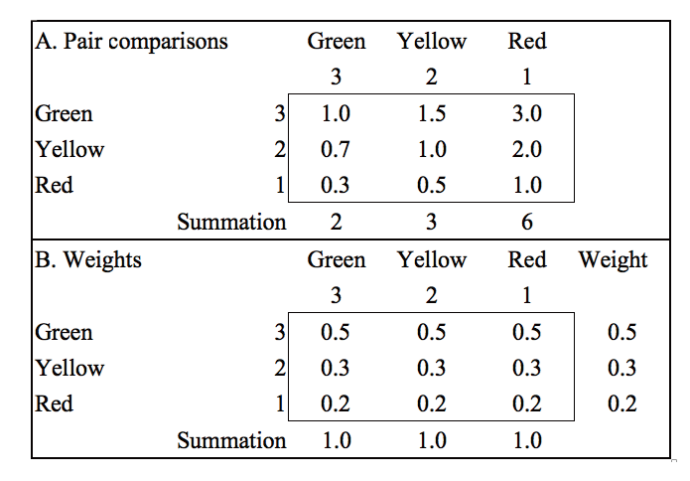
Calculation of weights for evaluating and ranking hospital performance. In step 1, scores are assigned from 3 (best, green) to 1 (worst, red). In step 2, pair comparison (eg, 3/2=1.5, 2/1=2, 1/3=0.3, etc) is performed to obtain the odds for each cell in the top panel. In step 3, the odds/summation ratio is calculated for each cell in the bottom panel, and the bottom row is averaged to obtain the final weight (eg, 0.5, 0.3, and 0.2).

We used the analytic hierarchical process [38] to calculate the weight for each category of performance and then determined the hospital type that performed best in the BCG growth/share matrix according to the following protocol: (i) calculating the percentage in the colorful cluster (ie, the degree of growth/share performance), (ii) multiplying the percentage by the performance weight (ie, 0.5, 0.3, and 0.2 in Figure 3 and the summation equal to 1.0), (iii) summing the weighted score for each hospital type, and (iv) selecting the hospital type that performs best in duplicative prescriptions. The details of the weight calculation are shown in Figure 3.

Finally, we used Kendall coefficient of concordance (*W*) [29,30] to evaluate whether the performance rankings were unanimous.

### Statistical Analysis

SPSS 19.0 for Windows (SPSS Inc, Chicago, IL, USA) and MedCalc 9.5.0.0 for Windows (MedCalc Software, Mariakerke, Belgium) were used to calculate Cronbach α, dimension coefficients, and other scale quality indicators used in this study. The cloud computation was programmed using the active server pages on the website (see Multimedia Appendix 3). MS Excel Visual Basic for Application (Microsoft Corporation, Redmond, WA, USA) was used to organize the study data.

## Results

### Data Quality Assessment

The scaling quality for the study data was found to be acceptable (dimension coefficient>0.67 and Cronbach α>.70), indicating that these duplicative prescription ratio data are reliable and consistent with our expectation (Table 2).

**Table 2:** Quality assessment of the study data.

Type of duplicative prescription	Dimension coefficient	Cronbach α (reliability)	Average variance extracted	Construct reliability
Antihypertension	0.69	.79	0.80	0.99
Antihyperglycemia	0.73	.91	0.85	0.99
Antihyperlipidemia	0.71	.88	0.75	0.98

### Building Online Dashboards

**Figure 4:**
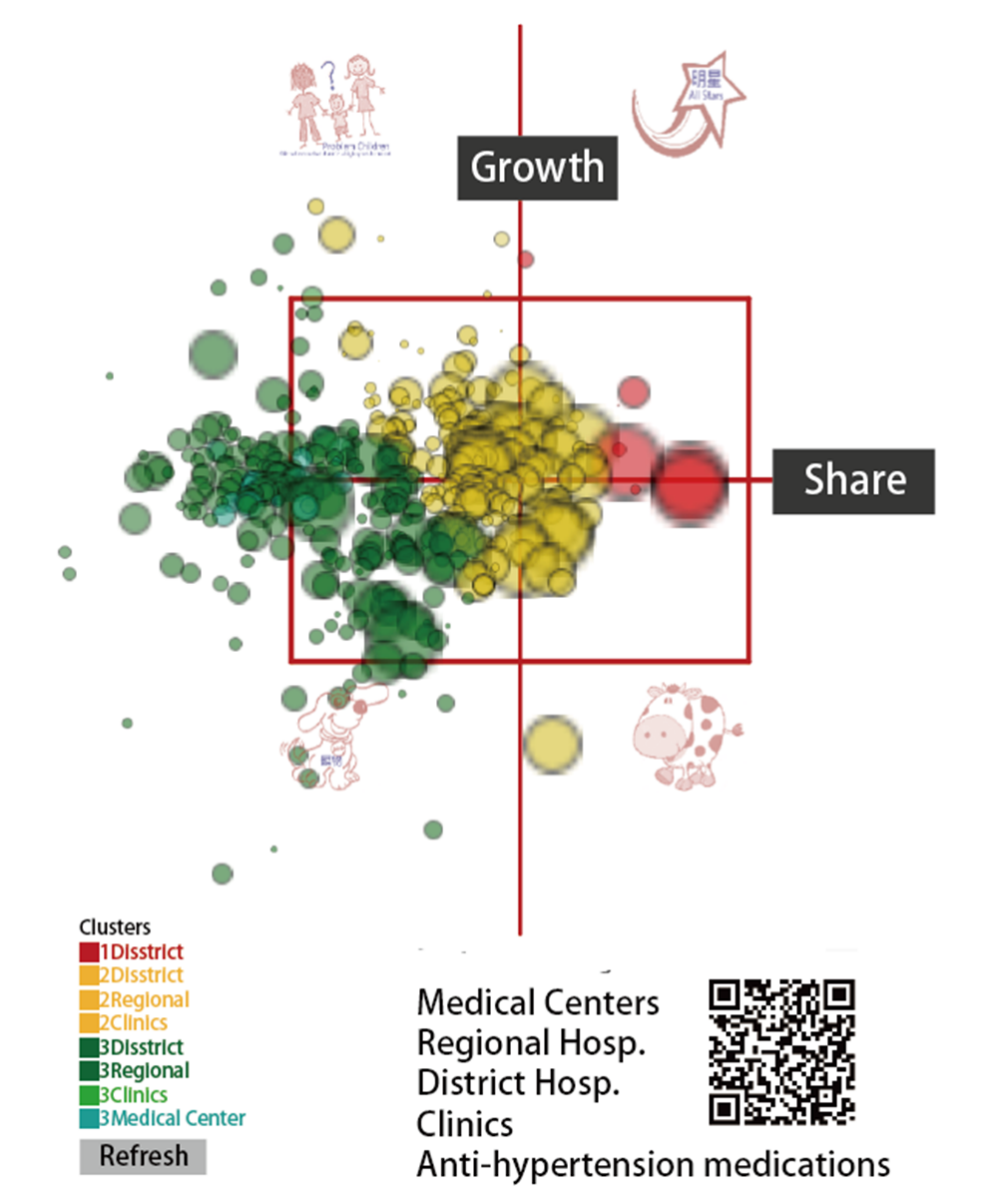
Dashboard of antihypertension duplicate prescription performance.

**Figure 5:**
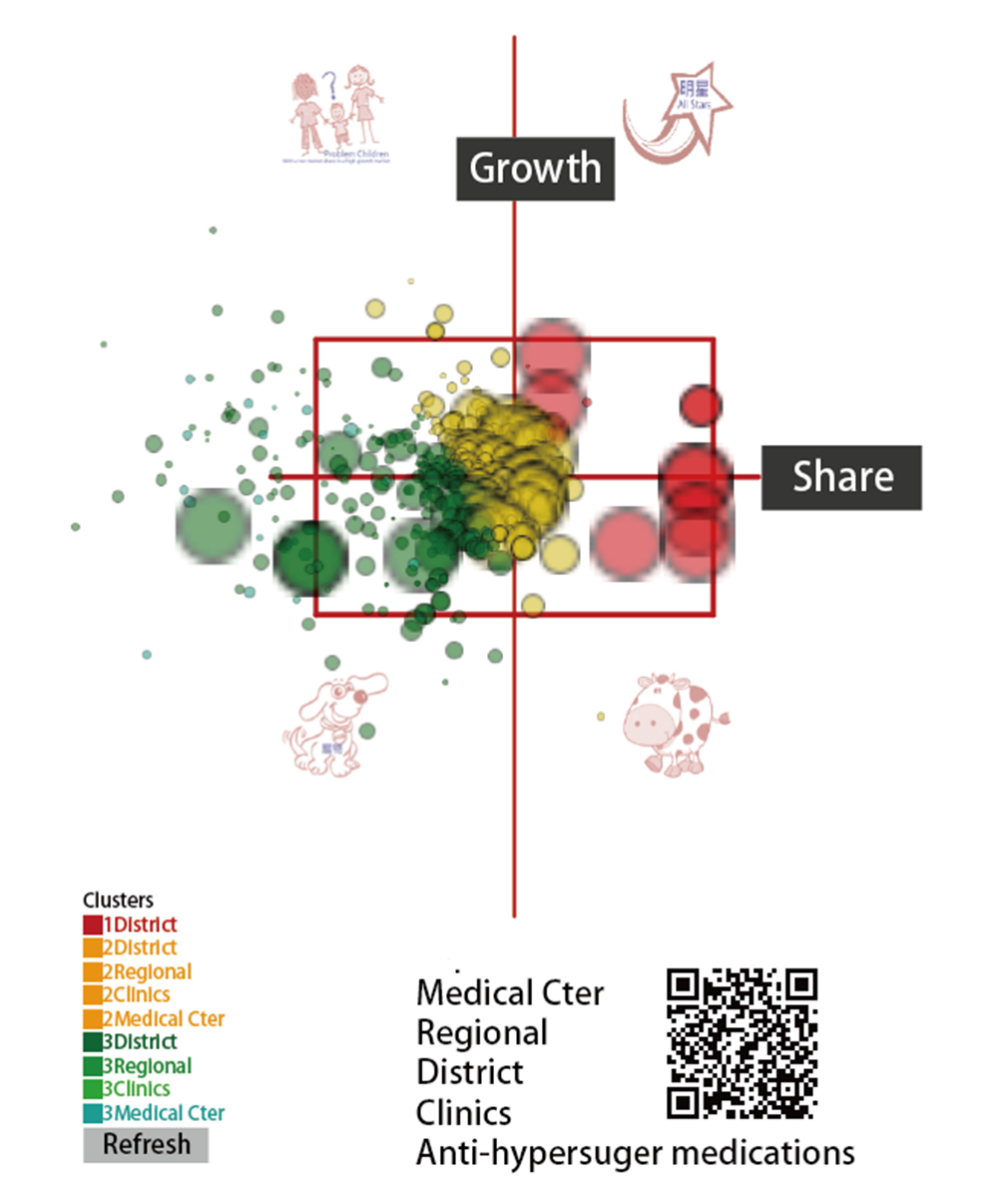
Dashboard of antihyperglycemia duplicate prescription performance.

**Figure 6:**
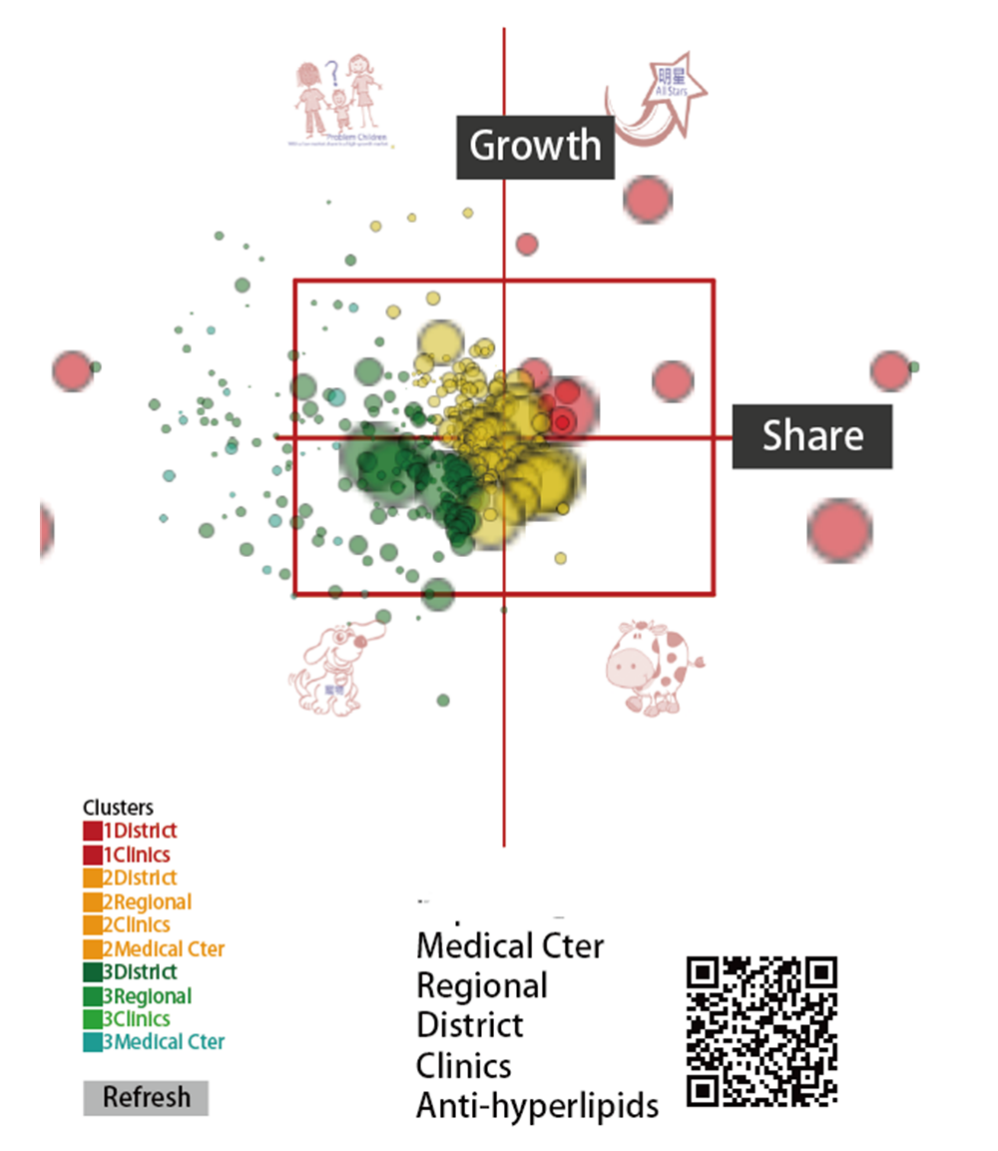
Dashboard of antihyperlipidemia duplicate prescription performance.

The dashboards shown in Figure 4, Figure 5, and Figure 6 show all of the hospitals on the respective maps for duplicative prescriptions of antihypertension, antihyperglycemia, and antihyperlipidemia, in which each hospital is appropriately colored and sized by a bubble. Clicking the bubble shows two kinds of control charts that indicate the traditional 2-year trend and recent 1-year moving average with a trend as illustrated in Figure 2. The control area is divided by the 2 SDs on the X and Y axes, facilitating examining any hospital with extreme performance outside the area. We can also click the icons on the bottom to view the partial type of hospital or the colorful cluster of interest in the left bottom panel. Interested readers may consult references [39-41] or scan the QR codes of the study duplicative prescriptions in Figures 4 to 6.

### Selecting the Best-Performing Hospital Type in Duplicative Prescription Management

As shown in Table 3, the frequency of hospitals in the BCG growth/share matrix on a dashboard showed inconsistent homogeneity among the hospital types, indicating that district hospitals are the largest in number with increasing growth and share (red color code). After summing the weighted scores for each type of hospital in each category of duplicative prescriptions (Table 4), it is clear that medical centers and regional hospital perform best in the growth/share matrix of duplicative prescriptions.

Kendall *W* was 0.781 (χ^2^_2_=4.67*,* sum of squares=14, *P*=.10), indicating that the rankings for different types of duplicative prescriptions were consistent (Table 4). Regional hospitals ranked first, demonstrating superiority to the medical centers in the duplicative prescription of antihyperlipidemia medications. Otherwise, Kendall *W* was 1.0 (Chi square_2_=6.0*,* *P*=0.05) if the regional hospitals also ranked second.

**Table 3:** Frequency of the three types of duplicative prescriptions in the four quadrants on the dashboards.

Prescription and hospital type		Red (weight=0.2), n (%)	Yellow (weight=0.3), n (%)		Green (weight=0.5), n (%)		N		Score		Chi square (df=4)		*P* value	
**Antihypertension,**											**64.13**		**<.001**	
	Medical Center	N/A^a^	N/A		20 (100)		20		50.0					
	Regional Hospital	N/A	1 (1)		76 (99)		77		49.8					
	District Hospital	8 (2)	170 (56)		127 (42)		305		38.2^b^					
	Total	8 (2)	171 (42)		223 (56)		402		N/A					
**Antihyperglycemia**												**69.91**		**<.001**
	Medical Center	N/A	1 (5)		19 (95)		20		49.0					
	Regional Hospital	N/A	6 (8)		73 (92)		79		48.4					
	District Hospital	13 (4)	156 (51)		139 (45)		308		38.6					
	Total	13 (3)	163 (41)		231 (56)		407		N/A					
**Antihyperlipidemia**											**64.92**		**<.001**	
	Medical Center	N/A	1 (5)		19 (95)		20		49					
	Regional Hospital	N/A	2 (3)		75 (97)		77		49.4					
	District Hospital	13 (5)	143 (56)		101 (39)		257		37.3					
	Total	13 (4)	146 (41)		195 (55)		354		N/A					

^a^N/A: not applicable.

^b^Score is calculated as: 38.2=(2%×0.2+56%×0.3+42%×0.5)×100.

**Table 4:** Rankings of hospital type for duplicative prescriptions.

Hospital type	Antihypertension	Antihyperglycemia	Antihyperlipidemia
Medical center	1	1	2
Regional hospital	2	2	1
District hospital	3	3	3

## Discussion

### Principal Findings

We used dashboards with an mHealth tool to create an animated dashboard that represents the hospital performance sheet of managing duplicative prescriptions in Taiwan. The data quality were acceptable and effectively reflected the reliability and construct validity. The online dashboards enabled easy and clear interpretation of duplicative prescriptions related to hospital performance using multidisciplinary functionalities, demonstrating a trend toward reducing duplicative prescriptions among all types of hospitals. Medical centers and regional hospitals exhibited better performance improvement for reducing duplicative prescriptions for the three types of controlled medications compared with district hospitals. Kendall *W* was 0.78, which indicated that the performance rankings were not unanimous.

### Contributions to the Field

Many researchers have published studies based on Google Maps [42-44]. Other studies focused on incorporating the dashboard into a health care report card [45-49], which is worth applying as an informative dashboard to health care settings. However, to our knowledge, this is the first study to build a quality report card as a dashboard, especially using Google Maps, from mHealth.

Making hospitals more transparent [20-22] does not only involve providing a static JPG-format picture but also should include a dynamic dashboard, particularly using a URL to display on mHealth tools for easy comparisons. The dashboards established using the Google Maps application program interface (API) to display health care report cards [46-49] are unique and promising advances in both academic and health care settings for ensuring patient safety against duplicative prescriptions. As such, many other quality-of-care indicators shown on the TNHIA website [50] should be used with an animated dashboard to compare hospital performance rather than traditional static digits or figures [51]. We hope that subsequent studies can report other types of research results using the Google Maps API in the future.

We also found that many district hospitals have incomplete (or missing) data on the ratio of duplicative prescriptions. The reason might be that many district hospitals are significantly affected by the global budget payment system, forcing them to terminate their businesses due to difficult operations in health services.

Management differentiation strategies [52] can be applied through the BCG matrix to review the product portfolio [36,37]. Figures 4 to 6 display the four quadrants derived on market growth (along the Y-axis), relative market share (along the X-axis, indicating the momentum in trend based on previous time points; see Figure 2), and complements of mascots, which are the merits of this study by presenting the BCG matrix with a dashboard on a map.

The use of weights that should sum to 1.0 (as illustrated in Figure 3) differs from the traditional method of performance assessment such as a Likert-type survey using ordinal scores to measure individual performance by summing all item scores with weights not equal to 1.0. We further applied Kendall *W* coefficient to examine whether the performances across all types of hospitals for the three types of drugs were unanimous, demonstrating that the performance rankings were not unanimous and the difference resulted from variation among the drug types.

### Implications and Areas for Improvement

#### Easy Way to Build an Animated Dashboard

Google Maps provides programmers with an API to incorporate coordinates with visual representations and build a dashboard-type report card. We demonstrated the process of creating HTML in the video of Multimedia Appendix 1, which is rarely provided in related research. Interested readers may consult references [39-41] for further details related to Figure 2.

#### Algorithm for Big Data

The TNHIA website [50] includes many quality-of-care indicators. Intervention is necessary to allow for the systematic collection and analysis of quality-of-care data to assess key quality indicators for all hospitals in a country (or in a region) and provide a “dashboard” feedback to hospitals. The moving control chart is superior to a conventional control chart by providing more valuable information to users. The hospitals with the problem children mascot indicate a readiness to grow. By contrast, the hospitals with the cash cow mascot imply a declining trend. According to the strength of the BCG growth/share matrix, the use of three clusters classified in different colors (red, yellow, and green) and four quadrants are unique and novel in the related literature.

#### Scale Quality Indicators

As mentioned above, the data quality should be ensured before analysis. This task involves examining the responses that are consistent and reproducible with acceptable reliability and validity [31,32]. Numerous indicators have been proposed to reflect the various ways in which data can be consistent and reproducible. In addition, Cronbach α is a necessary but not a sufficient component of validity [53,54]. Thus, in the present study, we applied other scale quality indicators, including dimension coefficient, average variance extracted, and construct reliability, to examine the quality of the dataset.

#### Strength of the Study

We evaluated the scale quality with several indicators based on classical test theory. Furthermore, we illustrated the importance of the API in Figure 1 and Multimedia Appendix 4 to demonstrate the infrastructure for applying big data in the cloud computation to build a dashboard-type report card. The BCG matrix incorporated with dashboards can be generalized to many other quality-of-care indicators in the future. The concept of moving control charts [54] can also be applicable and feasible for future use.

#### Limitations of the Study

Several issues should be considered thoroughly in the future. First, the study data were incomplete, especially for the district hospitals. Thus, inference making, such as for district hospitals with poor performance in controlling duplicative prescriptions, should be conservative. This limitation calls for further research and validation.

Many innovations have been introduced with advances in science and technology, such as the visual dashboard on Google Maps using the coordinates to display and line plots on cloud computation as shown in Figures 4 to 6. However, these achievements are not free of charge. For example, the Google Maps API requires a paid project key for use on the cloud platform, and the line plot also requires payment (to JPowered) for the template used on the website. Thus, the second limitation of the module is that it is not publicly accessible and is difficult to mimic by other authors or programmers for use in a short period of time.

Third, the mascots illustrated in the BCG matrix, such as stars, problem children, cash cows, and dogs, might be inappropriate in health care settings. Other mascots such as Santa Claus, productive cows, or dejected dogs, could refer to appropriate dashboard-type report cards in the future.

Fourth, the scaling quality for the study data was found to be acceptable (ie, dimension coefficient>0.67 and Cronbach α>.70), indicating that these duplicative prescription ratio data are reliable and consistent with our expectation. The dimension coefficients were relatively low (ie, 0.69, 0.71, and 0.73), indicating that all datasets were weak when measuring a one-dimensional feature (ie, duplicative prescriptions). Therefore, there is low confidence when using the result to make an inference for the future. Further studies should pay more attention to the issue of data fitting to the unidimensional requirement.

Fifth, the effect of weights was obvious due to different sample sizes in different hospital types. We normalized the summed weights to be 1.0 and ensured fair comparisons among hospital types across performance categories (ie, red, yellow, and green bubbles). If the percentages of the performance categories differ among hospital types, the weights will affect the assessment results. For this reason, we used an analytic hierarchical process [38] to calculate the weight for each category of performance and then determined the hospital type that performed best in the BCG growth/share matrix, which is worth noting for future assessments.

### Conclusion

This study provides a demonstrated platform with an online quality report card on detecting the performance of duplicative prescriptions to help health care practitioners easily upload data and quickly provide feedback on visual representations on an online dashboard. These dashboards can be used to build an online report card for hospitals under supervision of the public based on mHealth and uHealth in the future.

## Appendix

Multimedia Appendix 1 MP3: How to build Google maps for this study.

Multimedia Appendix 2 The moving average control chart used in this study.

Multimedia Appendix 3 Excel dataset.

Multimedia Appendix 4 MP3: How to manipulate the mHealth dashboard on Google Map.

## Abbreviations

API: application programming interface
ATC: Anatomical Therapeutic Chemical
BCG: Boston Consulting Group
HTML: hypertext markup language
mHealth: mobile health
SNA: social network analysis
TNHIA: Taiwan National Health Insurance Administration
